# Mitochondria-resident SBK3 confers protection against pressure overload-induced heart failure in mice

**DOI:** 10.3724/abbs.2025098

**Published:** 2025-07-18

**Authors:** Aihua Yang, Yuhang Wang, Yifeng Zhang, Xiaojun Wang, Yi Qian, Wenjing Zhao, Hongyan Qian, Jun Ren, Weizhong Zhu

**Affiliations:** 1 Department of Pharmacology School of Medicine and School of Pharmacy Nantong University Nantong 226019 China; 2 Affiliated Maternity and Child Health Care Hospital of Nantong University Nantong 226018 China; 3 Shanghai Institute of Cardiovascular Diseases Department of Cardiology Zhongshan Hospital Fudan University Shanghai 200032 China; 4 National Clinical Research Center for Interventional Medicine Shanghai 200032 China; 5 State Key Laboratory of Cardiovascular Diseases Zhongshan Hospital Fudan University Shanghai 200032 China

**Keywords:** heart failure, cardiac hypertrophy, mitochondria, SBK3, transverse aortic constriction

## Abstract

Pathological myocardial hypertrophy, often caused by hypertension, is a well-established independent risk factor for heart failure. *SBK3*, a gene selectively expressed at relatively high levels in cardiac tissues, has an unclear functional role in the heart. This study is designed to examine the role of SBK3 in transverse aortic constriction (TAC)-induced heart failure, aiming to identify a novel mitochondrion-targeted therapeutic strategy for heart failure. The subcellular localization of SBK3 in adult rat cardiomyocytes is investigated by western blot analysis and immunofluorescence staining, which reveal that SBK3 is located in the mitochondria. Subsequent western blot analysis shows that SBK3 protein expression is downregulated under pathological hypertrophy. To assess the functional relevance of this observation, SBK3 is overexpressed both *in vivo* (via cardiac-specific AAV9-cTNT) and *in vitro* (via adenoviral transduction). *In vitro*, adenovirus-mediated overexpression of SBK3 significantly inhibits ANP and BNP expression and increases the Ca ^2+^ transient amplitude in angiotensin II (Ang II)-induced hypertrophic cardiomyocytes. *In vivo*, cardiac-specific SBK3 overexpression using cTNT promoter-containing adeno-associated virus 9 inhibits TAC-induced cardiac hypertrophy and heart failure. Mechanistically, SBK3 exerts its cardioprotective effects by preserving the mitochondrial ultrastructure and regulating the balance of respiratory chain complexes. In addition, SBK3 modulates key regulators of mitochondrial dynamics, including fission and fusion proteins, thereby contributing to mitochondrial integrity and protection against pathological cardiac remodeling.

## Introduction

Cardiac hypertrophy is a common pathological feature of various cardiovascular diseases, including hypertension, and frequently precedes the development of heart failure, a condition of increasing public health significance [ 1– 3] . Among the different forms of hypertrophy, pressure overload–induced myocardial hypertrophy, such as that caused by aortic stenosis, is recognized as an independent risk factor for heart failure [4]. Despite significant advances in clinical management, the morbidity and mortality associated with heart failure remain high and continue to rise worldwide [ 5, 6] .

In the context of cardiovascular diseases, mitochondria serve not only as a source of cellular damage but also as a target for therapeutic intervention [7]. Changes in mitochondrial function are closely associated with cardiac hypertrophy and heart failure [8]. Evidence suggests that mitochondrial homeostasis in the myocardium is integral to cardiac health and is implicated in the progression of a broad spectrum of cardiac disorders. This includes disruption of the mitochondrial structure, dynamics, mitophagy, and energy metabolism [ 9– 11] . Interestingly, compensatory cardiac hypertrophy—a temporary adaptive response to increased workload—is often associated with the preservation of mitochondrial function [ 8, 12, 13] . Therefore, targeting mitochondrial function to prolong the physiological compensatory phase or reverse pathological hypertrophy may serve as a promising therapeutic strategy for hypertrophic heart failure.

SH3 domain binding kinase family member 3 (SBK3), also known as SGK110, is encoded on human chromosome 19 [14]. A genome-wide association study (GWAS) revealed that the SBK3 protein is predominantly enriched in heart tissues and is strongly associated with cardiovascular function [14]. SBK3 is closely related to SBK2, with the two genes located only a few kilobases apart. In 2022, van Gorp and colleagues [15] identified *SBK2* as a gene enriched in both atrial and ventricular tissues, where it contributes to sarcomere integrity. SBK3 has a spatial distribution similar to that of SBK2.

According to RNA abundance data from the Human Protein Atlas (HPA; https://www.proteinatlas.org), SBK3 is predominantly expressed in the heart and localized to mitochondria. However, its role in cardiac function remains poorly understood.

In this study, we utilized adeno-associated viral 9 (AAV9), which is known for its high efficiency and cardiac specificity, along with the cardiac-specific promoter cTNT to overexpress SBK3 in mouse hearts. This approach was used to investigate the role of SBK3 in transverse aortic constriction (TAC)-induced heart failure and its potential involvement in maintaining mitochondrial homeostasis.

## Materials and Methods

### Reagents and materials

Angiotensin II (HY-13948A) was obtained from MedChem Express (Shanghai, China). Medium 199, type II collagenase, and protease type XIV were obtained from Sigma-Aldrich (St Louis, USA). Laminin was purchased from Roche (Basel, Switzerland). Trypsin-EDTA solution, cell mitochondria isolation kits and protein assay kits were obtained from Beyotime (Shanghai, China). Anti-SBK3 (PA5-48684 for western blot analysis, PA5-85058 for immunofluorescence) antibody was obtained from Thermo Fisher Scientific (Waltham, USA). The total OXPHOS Rodent Cocktail antibody (ab110413) containing 5 mouse mAbs, the mitochondrial complexes I-V, was acquired from Abcam (Cambridge, UK). Anti-COX IV (4844), anti-Drp1 (8570), anti-phospho-Drp1 (3455), anti-mitofusin-1 (14739), anti-mitofusin-2 (9482), and anti-OPA1 (80471) antibodies were purchased from Cell Signaling Technology (Danvers, USA). Anti-β-tubulin (66240-1-Ig) and anti-Flag (20543-1-AP) antibodies were purchased from Proteintech (Wuhan, China). Hematoxylin-eosin (HE), Masson’s trichrome staining kits, wheat germ agglutinin (WGA) staining kits, and electron microscope fixation solutions were obtained from Servicebio (Wuhan, China). The calcium ion fluorescent probe Fluo-3AM (IF0150) was purchased from Solarbio (Beijing, China).

### Construction of viruses

The viruses used in the experiment were purchased from Jikai (Shanghai, China). An adenovirus vector (AD-SBK3) was constructed for SBK3 overexpression *in vitro*. The adeno-associated virus vector 9 of SBK3, driven by the cardiomyocyte-specific promoter cTNT, was used to ensure specific expression in heart tissue. The structural framework was AAV9-cTNT-SBK3-flag-GFP (AAV9-SBK3). The lentiviral vector system consists of three plasmids: the GV lentiviral vector series, pHelper 1.0 vector and pHelper 2.0 vector. Three RNAi target sequences were designed for the *SBK3* gene sequence to silence *SBK3* (Lenti-SBK3-RNAi). Their target sequences are as follows, NC-RNAi, 5′-TTCTCCGAACGTGTCACGT-3′; SBK3-RNAi(1), 5′-AAGCTAGGTTCCGGTTCCTAT-3′; SBK3-RNAi(2), 5′-TTGCTCGTTGGATGACCACTA-3′; and SBK3-RNAi(3), 5′-AAGCTTGGGAGGTGTATCCTA-3′.

### Animals and treatment

This study employed a modified TAC procedure that did not require a mechanical ventilator for the mice [16]. C57BL/6 mice were lightly anaesthetized with 1.5%–2% isoflurane and placed in the supine position on a heated platform at 37°C. The neck was exposed, and the underlying muscle was bluntly dissected using surgical forceps to expose the trachea. Using surgical scissors, a midline incision was made through the sternum to access the thoracic cavity. A 6-0 suture was passed between the innominate artery and the left common carotid artery, with a 27-G needle placed parallel to the aortic arch. The suture was tied securely around the needle, which was then gently removed to create the constriction. The chest and neck skin were subsequently sutured closed. Anaesthesia and body temperature were maintained throughout the procedure until full recovery.

Echocardiographic assessment was conducted under the same anaesthetic protocol. Long-axis M-mode images were acquired at a constant heart rate of 400–500 beats per minute.

All experimental procedures in animal research conformed to the guidelines for laboratory animal care and were approved by the Institutional Animal Care and Use Committee of Nantong University (approval number IACUC20210916-1001).

### Histological analysis

The mice were anaesthetized via an intraperitoneal injection of 0.5% pentobarbital sodium (0.1 mL/10 g). Left ventricular specimens were fixed with 4% paraformaldehyde and embedded in paraffin. Cardiac sections were then analyzed by histology and immunohistochemistry to assess cardiomyocyte hypertrophy and fibrosis. HE [17] and Masson trichrome staining (Masson) were used to evaluate the degree of cardiac fibrosis, whereas WGA staining was used to assess the cross-sectional area of cardiomyocytes.

### Transmission electron microscopy

A 1-mm ^3^ sample of mouse heart tissue was immediately placed in a Petri dish containing electron microscope fixation solution. The mixture was then fixed at room temperature for 2 h, transferred to an EP tube, and further fixed at 4°C. After gradient dehydration, the samples were permeated and polymerized overnight. An ultrathin film of 60–80 nm was subsequently cut and double-stained with 2% uranium acetate and 2.6% lead citrate. Following drying, the images were visualized via a transmission electron microscope (Hitachi, Tokyo, Japan).

### Serum biochemistry analyses

Serum levels of lactate dehydrogenase (LDH) and creatine kinase (CK) were determined via commercial kits (Jiancheng Ltd., Nanjing, China) according to the manufacturer’s instructions.

### Isolation and culture of rat cardiomyocytes

The adult rat cardiomyocytes (ARCMs) [17] and neonatal rat cardiomyocytes (NRCMs) [18] used in the experiment were isolated and cultured by enzymatic hydrolysis. ARCMs were cultured in M199 medium, while NRCMs were cultured in DMEM/F-12 medium. ARCMs were transfected with SBK3-overexpressing adenovirus dissolved in an enhancing solution for 8 h. After transfection, the ARCMs were cultured for a total of 60 h. NRCMs were transfected with lentivirus (Lenti-SBK3-RNAi) to silence *SBK3*, after which the medium was replaced by fresh DMEM/F-12. NRCMs were cultured for 72 h.

### Cytosol-mitochondria isolation

Isolated adult rat cardiomyocytes were seeded into culture plates and allowed to adhere. After 2 h, the medium was replaced, and non-adherent cardiomyocytes were removed. Cardiomyocytes were washed once with PBS, and mitochondrial isolation buffer containing PMSF (added just before use) was added. Cardiomyocytes were placed on ice and gently scraped off after 10–15 min. The cardiomyocyte suspension was transferred to a suitable glass homogenizer and homogenized 10–30 times.

The homogenate was subsequently centrifuged at 600 *g* for 10 min at 4°C to remove debris and nuclei. The resulting supernatant was carefully transferred to a new centrifuge tube and centrifuged at 11,000 *g* for 10 min at 4°C. The supernatant obtained from this step represented the cytoplasmic fraction, while the pellet contained the mitochondria isolated from cardiomyocytes [19].

### Measurement of Ca ^2+^ transients in cardiomyocytes

Isolated ARCMs were inoculated onto circular coverslips and cultured in 24-well plates under standard conditions (37°C with 5% CO _2_). Cardiomyocytes were washed three times with Tyrode solution (NaCl 140 mM, KCl 5.4 mM, MgCl _2_ 1 mM, HEPES 5 mM, glucose 5.5 mM, CaCl _2_ 1.0 mM, pH 7.4 adjusted with NaOH) at room temperature. Tyrode’s solution containing Fluo-3 AM (10 μM) was added, and the cardiomyocytes were incubated at 37°C for 20 min. After three times wash with Tyrode’s solution, coverslips containing cardiomyocytes were placed in an electric field-stimulated perfusion bath for continuous stimulation (0.5 Hz, 10 V) [20]. Changes in fluorescence were simultaneously recorded using a confocal microscope (Leica, Wetzlar, Germany) at an excitation wavelength of 485 nm. Each sample was visualized for approximately 8 s.

### Immunofluorescence staining

Cardiomyocytes were washed, fixed, permeabilized, and blocked for 10 min each. The primary antibody was added, and the samples were incubated overnight at 4°C. On the second day, the primary antibody was discarded, and the secondary antibody was added and incubated at room temperature for 2 h. After the nuclei were stained with DAPI, the samples were sealed with a fluorescence quencher and observed under a confocal microscope [21]. When necessary, MitoTracker dye was used for mitochondrial localization.

### Phalloidin staining

Following fixation and permeabilization, pre-prepared phalloidin working solution (100 nM) was added to the cardiomyocyte preparations, which were subsequently incubated for 30 min at room temperature in the dark. After the samples were washed with PBS, Hoechst stain was applied, and the samples were incubated for an additional 10 min under the same conditions. Anti-fade mounting medium was then used to seal the samples. Phalloidin-stained actin filaments were subsequently observed and analyzed using a fluorescence microscope [22].

### qPCR analysis

Total RNA was extracted via the FastPure Cell/Tissue Total RNA Isolation kit (Thermo Fisher Scientific). Reverse transcription was performed with the PrimeScriptTM RT reagent kit (Thermo Fisher Scientific). Then, using *GAPDH* as an internal reference gene, cDNA was amplified using SYBR Green reagents (Takara, Shiga, Japan). Analysis was performed using the 2 ^−ΔΔCt^ method [23]. The sequences of all the primers (Sangon Biotech, Shanghai, China) used are listed in Table 1.

**Table:** **Table 1** Sequences of primers used in real-time PCR

Gene	Species	Forward primer (5′→3′)	Reverse primer (5′→3′)
*ANP*	Mouse	GAGAAAGATGCCGGTAGAAGA	AAGCACTGCCGTCTCTCAGA
*BNP*	Mouse	CTGCTGGAGCTGATAAGAGA	TGCCCAAAGCAGCTTGAGAT
*SBK3*	Mouse	GCGGCTAGTGGAGCTAACAG	AGGGTCTGTAGTAGGCCTGG
*GAPDH*	Mouse	AGTCCCTGCCCTTTGTACACA	CGATCCGAGGGCCTCACTA
*α-SMA*	Mouse	ATCAGGGAGTAATGGTTGGAATGGG	CAGTTGGTGATGATGCCGTGTTC
*Col1a1*	Mouse	ACAGGCGAACAAGGTGACAGAG	AGGAGAACCAGGAGAACCAGGAG
*ANP*	Rat	GAGAGTGAGCCGAGACAGCAAAC	GGAAGAAGCCCTTGGTGATGGAG
*BNP*	Rat	AGTCTCCAGAACAATCCACGATGC	GCCTTGGTCCTTTGAGAGCTGTC
*GAPDH*	Rat	AAATGGTGAAGGTCGGTGTGAAC	CAACAATCTCCACTTTGCCACTG

### Western blot analysis

Proteins were extracted from isolated heart tissues or rat cardiomyocytes, separated by sodium dodecyl sulphate-polyacrylamide gel electrophoresis (SDS-PAGE), and subsequently transferred onto polyvinylidene difluoride (PVDF) membranes. The membranes were incubated with primary antibodies at 4°C overnight. The next day, the membranes were incubated with fluorescent secondary antibodies for 2 h at room temperature. Images were acquired by scanning with a fluorescence developer and analyzed via ImageJ [24].

### Statistical analysis

Data are presented as the mean ± SEM. Statistical analyses were performed via GraphPad Prism. Significance was analyzed using one-way ANOVA followed by the Student-Newman-Keuls test or two-way ANOVA, where appropriate. *P* value less than 0.05 was considered statistically significant.

## Results

### The mitochondrial protein SBK3 is downregulated following Ang II administration

**Figure 1:**
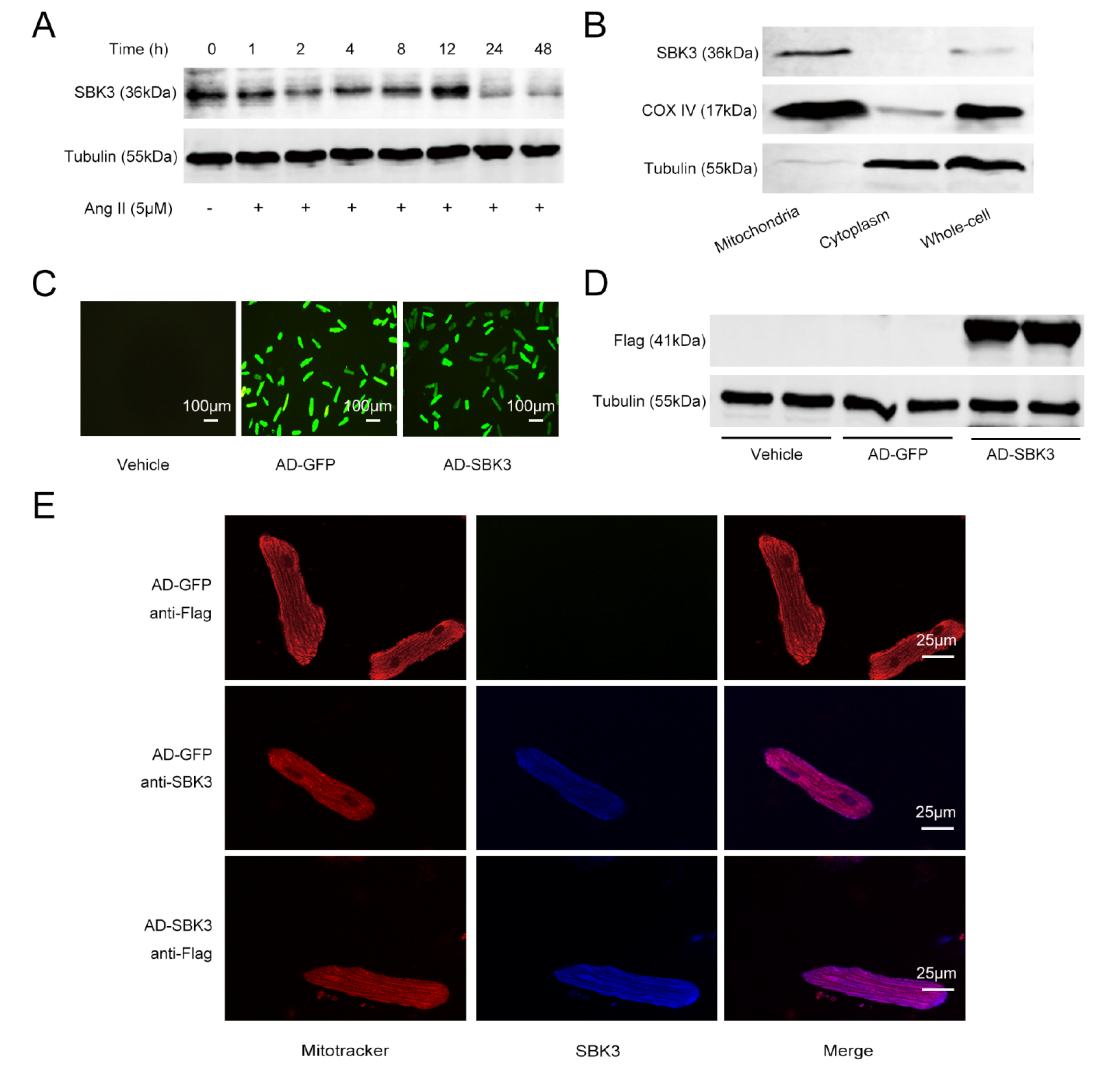
Figure 1 The mitochondrial protein SBK3 is downregulated following Ang II administration (A) The expression of SBK3 dynamically fluctuated following Ang II treatment and was downregulated after 24 h in adult rat cardiomyocytes (ARCMs), as detected by western blot analysis (n = 3). (B) SBK3 was successfully detected by western blot analysis in the mitochondria of rat cardiomyocytes but not in the cytoplasm (n = 3). (C,D) AD-SBK3 was successfully transfected into ARCMs, as evidenced by (C) fluorescence microscopy (scale bar = 100 μm) and (D) western blot analysis. (E) SBK3 was localized in the mitochondria of ARCMs, as detected by immunofluorescence staining; scale bar = 25 μm.

To investigate the role of SBK3 in the heart, adult rat cardiomyocytes were stimulated with Ang II (5 μM). Western blot analysis was performed at different time points to detect the expression of SBK3 following Ang II treatment. SBK3 expression exhibited dynamic changes and was significantly downregulated after 24 h ( Figure 1A).

To determine the subcellular localization of SBK3 in cardiomyocytes, western blot and immunofluorescence analyses were conducted using organelle-specific markers. The western blot results revealed that SBK3 was predominant in the mitochondrial fraction, with no detectable expression in the cytoplasmic fraction (mitochondria-depleted) ( Figure 1B). AD-SBK3 was successfully transfected into ARCMs, as evidenced by fluorescence microscopy and western blot analysis ( Figure 1C,D). Immunofluorescence analysis further confirmed that both exogenous and endogenous SBK3 were localized to the mitochondria, which was consistent with the western blot results ( Figure 1E). This study provides the first demonstration of the mitochondrial localization of SBK3 in cardiomyocytes.

### Overexpression of SBK3 suppresses Ang II-induced hypertrophy in rat cardiomyocytes

**Figure 2:**
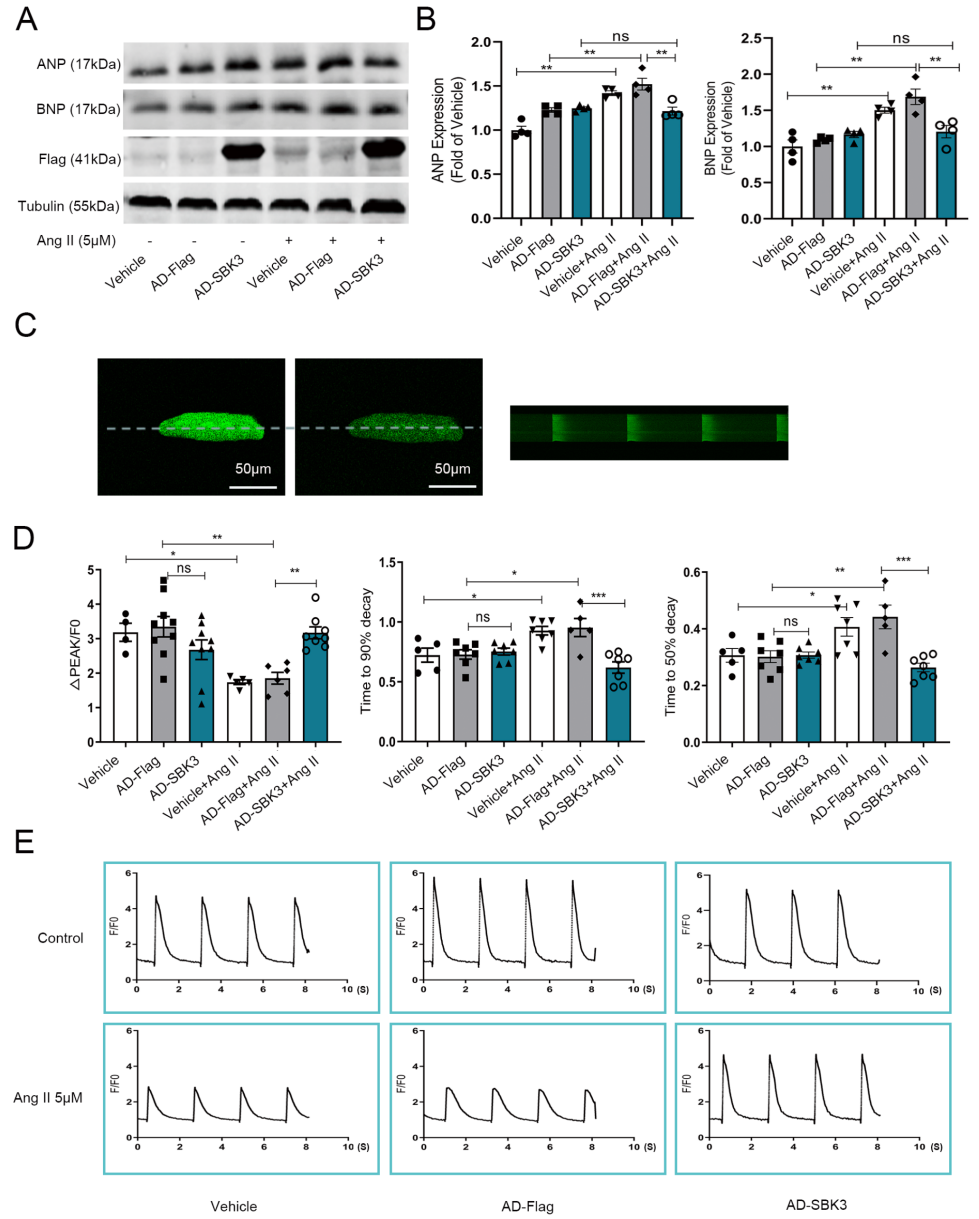
Figure 2 Overexpression of SBK3 suppresses Ang II-induced hypertrophy in rat cardiomyocytes (A) Representative images and (B) quantitative analysis of ANP and BNP protein expressions detected by western blot analysis in adult rat cardiomyocytes after treatment with 5 μM Ang II for 24 h (n = 4). Ca2+ transients were visualized via confocal microscopy. (C) Representative images of peak values, valley values, and line scans of Ca2+ fluorescence intensity are shown from left to right; scale bar = 50 μm. (D) Effects of SBK3 overexpression on the peak Ca2+ fluorescence intensity (△Peak/F0) and decay time of ARCMs induced by Ang II. (E) Schematic representation of the fluorescence intensity (F/F0) vs time (s) in each experimental group. Data are expressed as the mean ± SEM. *P < 0.05, **P < 0.01, ***P < 0.001, ns: not significant; one-way ANOVA test, followed by the Student-Newman-Keuls test.

Adenovirus-mediated overexpression of SBK3 was performed in adult rat cardiomyocytes challenged with Ang II (5 μM) to explore its potential cardioprotective role. As shown in Figure 2A,B, Ang II significantly upregulated the expressions of the pathological cardiac hypertrophy markers ANP and BNP, whereas SBK3 overexpression markedly reduced their expression levels. To assess the functional consequences, the calcium transient amplitude was measured via the calcium ion probe Fluo-3 AM under electric field stimulation (10 V and a frequency of 0.5 Hz) to mimic physiological excitation-contraction coupling. Ang II treatment led to a notable reduction in the Ca ^2+^ transient amplitude after 24 h, as shown in Figure 2C–E. SBK3 overexpression significantly restored the Ca ^2+^ transient amplitude ( ^△^Peak/F0) and shortened the duration of fluorescence decay in Ang II-stimulated hypertrophic cardiomyocytes, indicating improved calcium handling in hypertrophic cardiomyocytes.

**Figure 3:**
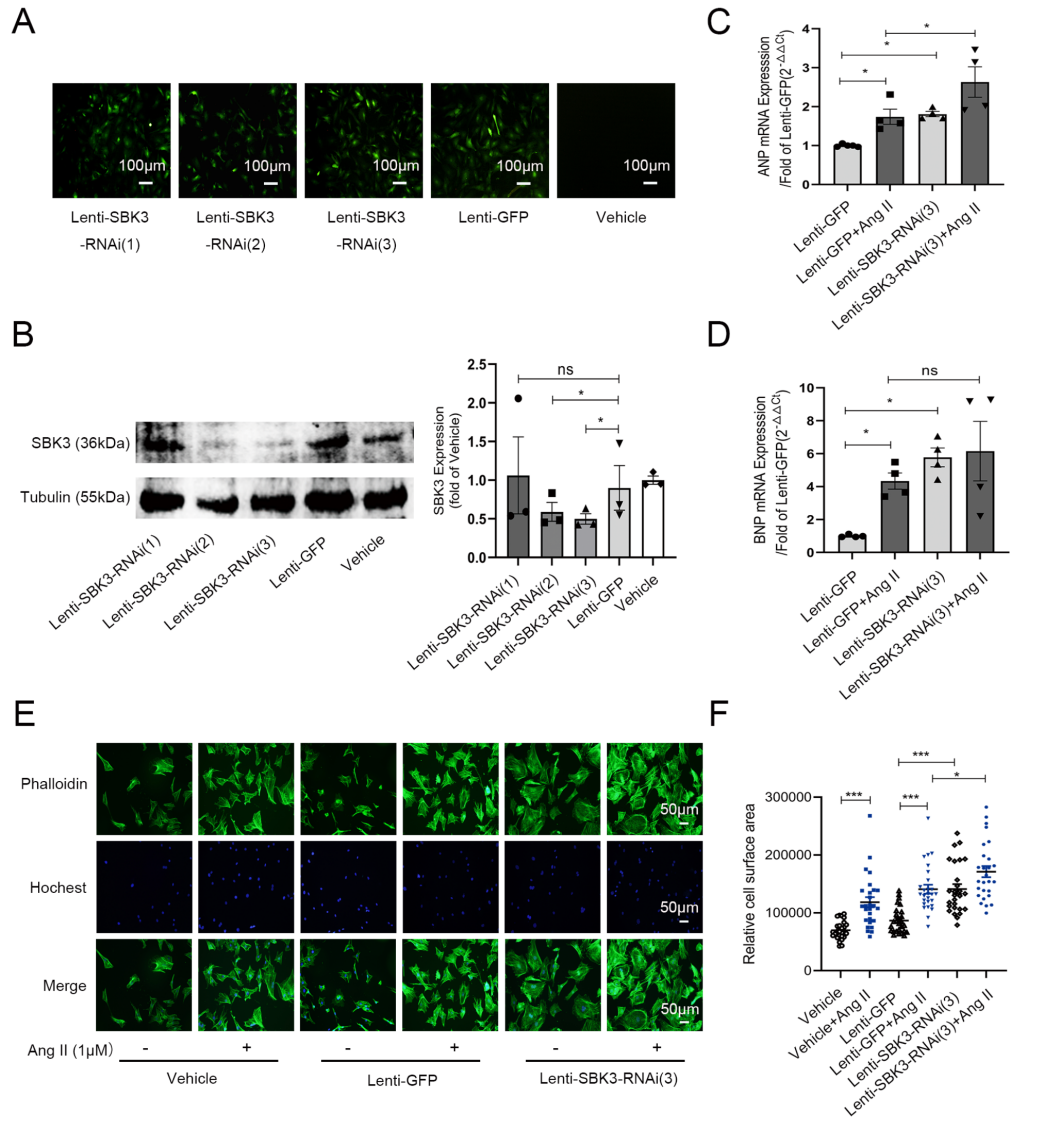
Figure 3 *SBK3* knockdown aggravates Ang II-evoked hypertrophy in rat cardiomyocytes (A) Fluorescence microscopy was used to detect the effect of Lenti-SBK3-RNAi in neonatal rat cardiomyocytes (scale bar = 100 μm). (B) The content of SBK3 in rat cardiomyocytes transfected with Lenti-SBK3-RNAi was detected by western blot analysis, n = 3. (C,D) Effects of SBK3 knockdown on the mRNA levels of ANP and BNP in angiotensin II-evoked cardiomyocytes were detected by RT-PCR, n = 4. (E) Hypertrophic areas of cardiomyocytes in NRCMs were detected by immunofluorescence staining (phalloidin staining, scale bar = 50 μm). (F) Quantitative analysis of the cardiomyocyte surface area. The average value of 10 cardiomyocytes in each visual field was taken as independent data. In total, 9 fields of view were taken from each group, and three parallel experiments were performed, n = 27. Rat cardiomyocytes were treated with 1 μM Ang II for 24 h. Data are expressed as the mean ± SEM. *P < 0.05, **P < 0.01, ***P < 0.001, ns: not significant; one-way ANOVA test, followed by the Student-Newman-Keuls test.

To further investigate the role of SBK3 in Ang II-induced cardiomyocyte hypertrophy, *SBK3* was silenced via siRNA. As shown in Figure 3A,B. Effective knockdown of *SBK3* via lenti-RNAi aggravated Ang II-induced increases in *ANP* and *BNP* mRNA levels ( Figure 3C,D), as well as cardiomyocyte hypertrophy ( Figure 3E,F). These findings suggest that SBK3 exerts a protective effect against Ang II-induced pathological hypertrophy by modulating hypertrophic gene expression and calcium homeostasis.

### Overexpression of SBK3 in the heart reduces mortality in TAC-treated mice

**Figure 4:**
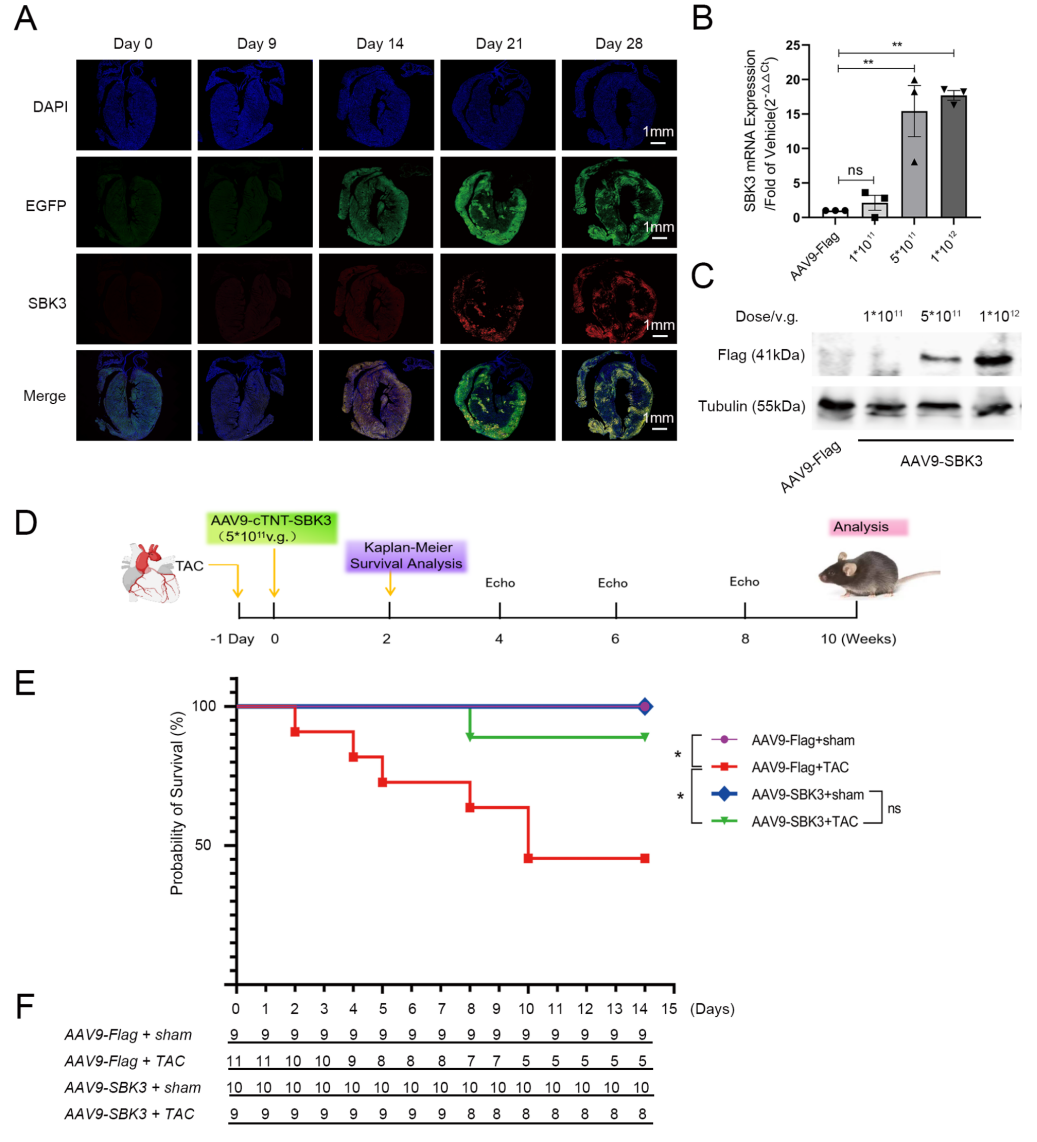
Figure 4 Overexpression of SBK3 in the heart via the AAV9 vector reduces mortality in mice subjected to TAC (A–C) AAV9-SBK3 was successfully expressed in mouse hearts, as determined by (A) immunofluorescence staining of tissues, (B) qPCR analysis (n = 3) and (C) western blot analysis. (D) A schematic diagram of the experimental program. (E) Kaplan-Meier survival analysis was conducted to evaluate the impact of SBK3 overexpression on the survival rate of TAC mice. (F) The actual number of living mice, n = 9–11; the AAV9-Flag + TAC group was supplemented with three additional mice due to high mortality. However, these additional deaths were not included in the overall death statistics. Data are expressed as the mean ± SEM. *P < 0.05, **P < 0.01, ns: not significant; one-way ANOVA test or Kaplan-Meier survival analysis.

Further *in vivo* investigations of the role of SBK3 in the heart are essential. To this end, we constructed an adeno-associated virus 9 (AAV9-SBK3) vector expressing SBK3 driven by the cardiomyocyte-specific promoter cTNT. The successful and stable expression of AAV9-SBK3 in mouse heart tissue was confirmed by immunofluorescence staining ( Figure 4A), qPCR analysis ( Figure 4B) and western blot analysis ( Figure 4C).

To evaluate the functional impact of SBK3 in pressure overload-induced heart failure, we utilized a TAC mouse model, which mimics human conditions such as hypertension or aortic valve stenosis leading to chronic heart failure (CHF). The experimental mice were randomly assigned to undergo either TAC or sham surgery (sham). On the following day, AAV9-SBK3 was administered via tail vein injection at a viral dose of 5 × 10 ^11^ v.g. per mouse. Cardiac function and ventricular wall thickness were monitored by echocardiography every two weeks for a total of 10 weeks ( Figure 4D).

Kaplan-Meier survival analysis was performed at 2 weeks post-surgery ( Figure 4E,F). The survival rate of the TAC-treated mice was 45.5%, whereas that of the sham-treated mice was 100%. Furthermore, the survival rate of TAC mice receiving AAV9-SBK3 was significantly greater (88.9%) than that of the control virus group (AAV9-Flag + TAC). These findings indicate that SBK3 markedly enhances survival in mice subjected to TAC.

### Overexpressing SBK3 in the heart suppresses cardiac hypertrophy and myocardial fibrosis in mice subjected to TAC

**Figure 5:**
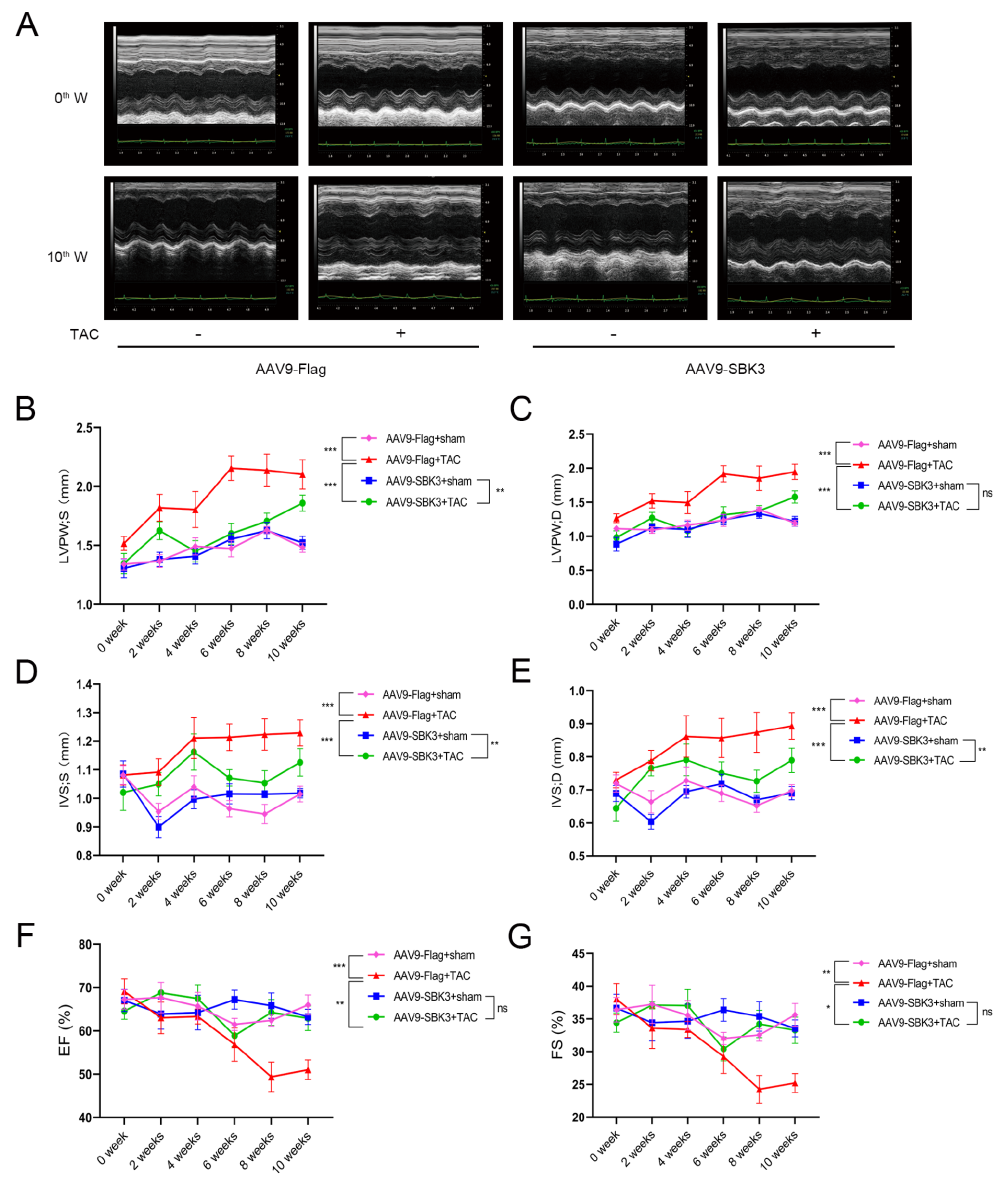
Figure 5 Overexpression of SBK3 in the heart restores cardiac function in mice subjected to TAC (A) Representative images of M-mode echocardiography in mice. (B–G) Mice underwent M-mode echocardiography every two weeks to evaluate (B,C) left ventricular posterior wall (LVPW) thickness, (D,E) interventricular septal (IVS) thickening at the diastole and systole, (F) ejection fraction (EF), and (G) fractional shortening (FS). Data are expressed as the mean ± SEM. *P < 0.05, **P < 0.01, ***P < 0.001, ns: not significant; two-way ANOVA.

The echocardiographic results at week 10 revealed significant thickening of the left ventricular posterior wall (LVPW) and ventricular septum (IVS) in the TAC group compared with the sham group ( Figure 5A–E), indicating cardiac hypertrophy. Additionally, TAC mice exhibited a marked decrease in EF and FS ( Figure 5F,G), with the EF decreasing below 50% and the FS decreasing below 25% by week 8, confirming the onset of heart failure. SBK3 overexpression attenuated IVS and LVPW thickening in TAC mice, prevented decreases in EF and FS, and partially restored cardiac function in TAC mice.

**Figure 6:**
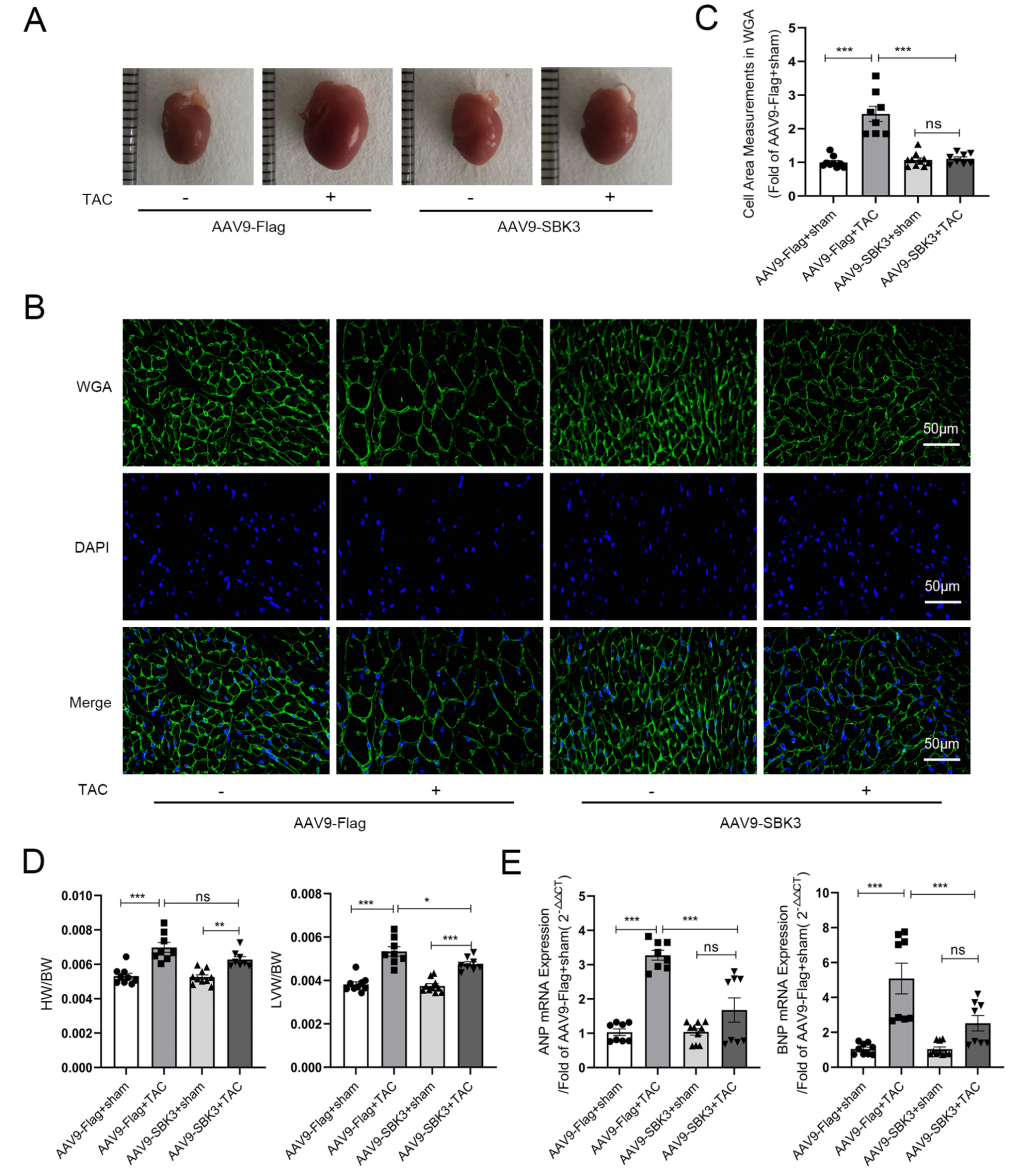
Figure 6 Overexpression of SBK3 in the heart suppresses cardiac hypertrophy in mice subjected to TAC (A–E) Overexpression of SBK3 in the heart inhibited TAC-induced hypertrophy, as evidenced by (A) representative photographs of mouse hearts (scale bar = 1 mm), (B) representative images of the cross-sectional area of cardiomyocytes (scale bar = 50 μm) and (C) quantitative analysis, (D) HW/BW ratio, LVW/BW, and mRNA expression of (E) ANP and BNP in mice challenged with Ang II (1000 ng/kg/min). n = 8–10 mice per group. Data are expressed as the mean ± SEM. *P < 0.05, **P < 0.01, ***P < 0.001, ns: not significant; one-way ANOVA test, followed by the Newman-Keuls test.

**Figure 7:**
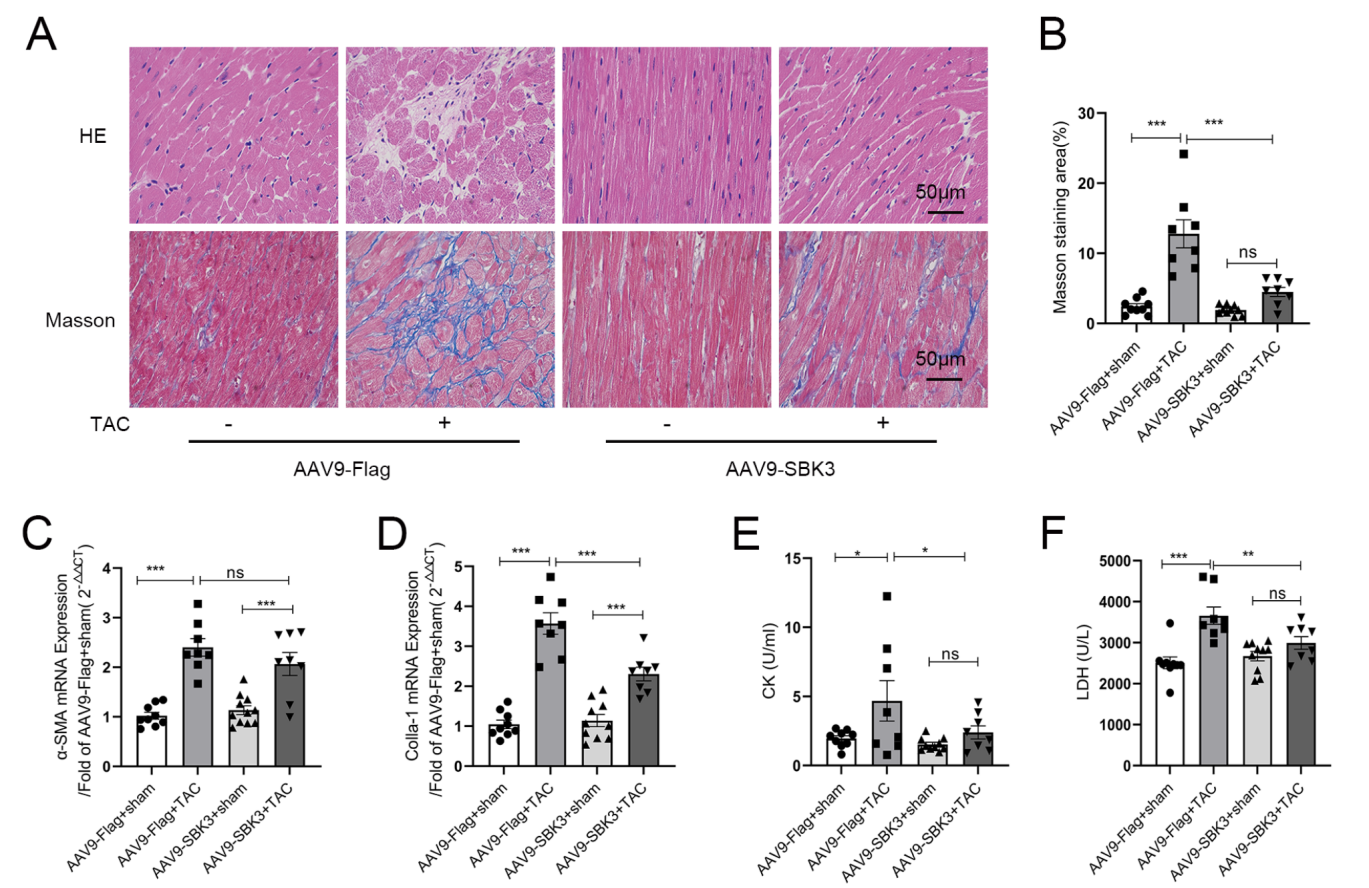
Figure 7 Overexpression of SBK3 in the heart suppresses myocardial fibrosis in mice subjected to TAC (A–F) Overexpression of SBK3 in the heart inhibited TAC-induced myocardial fibrosis, as evidenced by (A,B) Masson’s trichrome staining (scale bar = 50 μm) and quantitative analysis of the mRNA expressions of (C) α-SMA and (D) Colla-1 and the levels of (E) CK and (F) LDH; n = 8–10 mice per group. Data are expressed as the mean ± SEM. *P < 0.05, **P < 0.01, ***P < 0.001, ns: not significant; one-way ANOVA test, followed by the Newman-Keuls test.

Notably, AAV 9-SBK 3 significantly reduced the heart size ( Figure 6A) and the cardiomyocyte cross-sectional area ( Figure 6B,C), decreased the left ventricular weight-to-body weight ratio (LVW/BW) ( Figure 6D), and lowered the *ANP* and *BNP* mRNA levels ( Figure 6E) in TAC mice, indicating effective suppression of TAC-induced cardiac hypertrophy. Masson staining ( Figure 7A,B), along with α-SMA ( Figure 7C), Colla-1 mRNA ( Figure 7D), and the myocardial injury markers CK ( Figure 7E) and LDH ( Figure 7F), demonstrated that TAC induced significant myocardial injury and fibrosis. AAV9-SBK3 treatment significantly decreased CK and LDH levels in TAC mice and markedly reduced collagen deposition in cardiac tissues. These findings indicate that SBK3 overexpression mitigates myocardial injury and reduces myocardial fibrosis in TAC-treated mice.

### Overexpressing SBK3 in the heart rebalances mitochondrial homeostasis in TAC-treated mice

**Figure 8:**
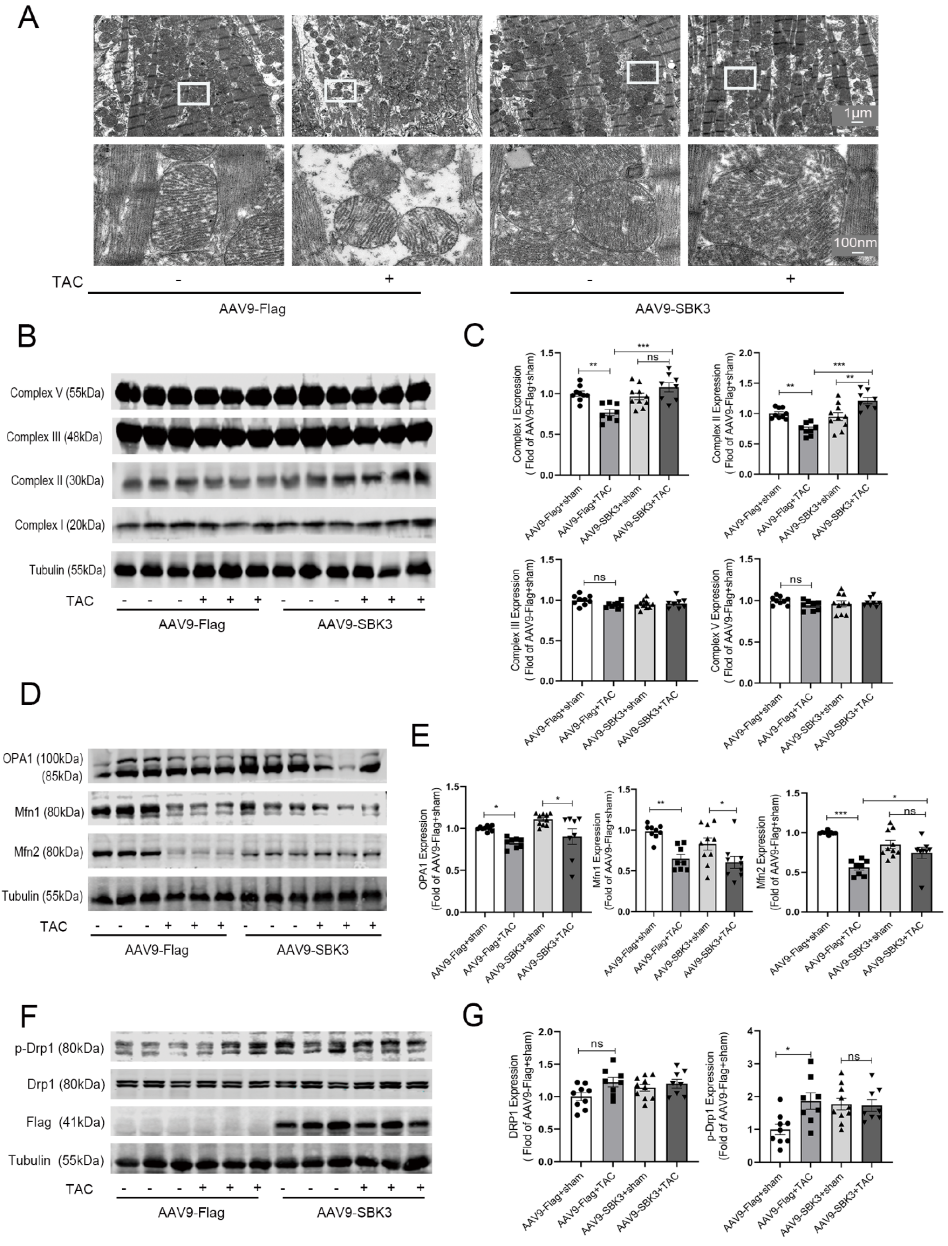
Figure 8 AAV9-SBK3 is beneficial for maintaining the morphology and function of mitochondria in mice subjected to TAC (A) Representative mitochondrial transmission electron microscopy images of cardiac tissue from each mouse group. (B–G) Mitochondrial respiratory chain complexes I–V (B: representative images of western blot analysis, C: quantitative analysis), OPA1, Mfn1, Mfn2 (D: representative images, E: quantitative analysis), and Drp1, p-Drp1 (F: representative images, G: quantitative analysis) were rebalanced following SBK3 overexpression in mice subjected to TAC. NADH-Q oxidoreductase (complex I), succinate-Q oxidoreductase (complex II), UQ-cytochrome C oxidoreductase (complex III), ATP synthetase (complex V). n = 8–10 mice per group. Data are expressed as the mean ± SEM. *P < 0.05, **P < 0.01, ***P < 0.001, ns: not significant; one-way ANOVA test followed by the Newman-Keuls test.

To assess the impact of SBK3 on mitochondrial function in TAC-induced heart failure, mitochondrial morphology was first examined by transmission electron microscopy. As shown in Figure 8A, TAC surgery led to disorganized and swollen mitochondria with disrupted cristae and structural damage. However, overexpression of SBK3 notably restored the integrity and arrangement of cristae, resulting in regular morphological features, tightened arrangement, and increased numbers of mitochondria. These findings suggested that SBK3 overexpression partially rescued mitochondrial ultrastructural disturbances in TAC-induced heart failure.

The proliferation of mitochondria under pathological circumstances does not increase ATP production [25]. In our study, TAC mice presented reduced protein expression of electron transport chain complexes ( Figure 8B,C), downregulation of the mitochondrial fusion proteins Mfn1 and Mfn2 ( Figure 8D,E), and increased phosphorylation of the mitochondrial fission protein DRP1 at S616 ( Figure 8F,G), a key event in the progression from cardiac hypertrophy to failure [ 26, 27] . AAV9-SBK3 restored the expressions of mitochondrial complex I, complex II and Mfn2 while also suppressing the phosphorylation of Drp1 at S616, which was reported to be overtly elevated in heart failure [ 28, 29] . These results highlight the protective role of SBK3 in preserving mitochondrial dynamics and function under pressure overload-induced stress.

## Discussion

Early-stage cardiac hypertrophy typically arises in response to elevated systemic pressure. Over time, this compensatory phase progresses to decompensation, which is marked by ventricular dilation, collagen deposition, and cardiomyocyte apoptosis, ultimately culminating in heart failure [ 30, 31] . Emerging evidence suggests that early intervention prior to the onset of heart failure may be key to halting disease progression [30].

In a recent study, deletion of the transcription factor Scleraxis reduced pressure overload-induced mortality in mice from 33% to 0% without affecting the degree of cardiac hypertrophy [32]. In our study, ventricular wall and ventricular septum thickness significantly increased as early as week 2 in TAC mice, whereas the ejection fraction remained preserved, indicating a compensatory hypertrophic phase. By week 8, EF declined, marking the transition towards heart failure. Notably, although ventricular wall and septal thickness remained elevated in TAC mice overexpressing SBK3 (compared with AAV9-SBK3 + sham), the ejection fraction remained within the normal range, and survival improved significantly. These findings are consistent with the *in vitro* findings that the maintenance of high SBK3 expression levels significantly inhibited Ang II-induced cardiomyocyte hypertrophy. Conversely, knockdown of *SBK3* markedly exacerbated Ang II-mediated hypertrophic responses in cardiomyocytes. These findings identify SBK3 as a novel, cardioprotective mitochondrial protein that may prevent the transition from compensated hypertrophy to heart failure, potentially by preserving myocardial mitochondrial homeostasis. Thus, SBK3 represents a promising therapeutic target for early intervention in hypertrophic heart disease.

Disruption of mitochondrial homeostasis is closely linked to the development of cardiac hypertrophy and heart failure [ 33, 34] . Stimuli such as Ang II, α- and/or β-adrenergic receptor activation, and pressure or volume overload [ 35, 36] have all been shown to impair mitochondrial function [ 37, 38] . Mitochondrial dynamics, in turn, regulate the distribution and stability of mtDNA nucleoids, impacting mitochondrial performance, the cellular energy balance, reactive oxygen species (ROS) production, and Ca ^2+^ signaling and ultimately governing cell fate, such as autophagy and apoptosis [ 39– 41] .

One key mechanism involves CaMKII-mediated phosphorylation of mitochondrial fission protein and Drp1 at the S616 site, which increases the frequency of mPTP opening, leading to mitochondrial damage and cardiomyocyte death [42]. Blocking Drp1 S616 phosphorylation or inhibiting Drp1 activity can prevent CaMKII-induced mPTP opening, cardiomyocyte death and myocardial hypertrophy. Notably, increased phosphorylation of Drp1 at S616 is observed in failing human hearts [29] and is commonly associated with mitochondrial autophagy [43]. Therefore, targeting Drp1 has emerged as a promising strategy for cardioprotection, for example, through the inhibition of Drp1-dependent mitochondrial autophagy via the use of neuraminidase 1 inhibitors to protect against adriamycin-induced cardiotoxicity [44].

In summary, our current study demonstrated that TAC-induced heart failure disrupts mitochondrial structure, reduces mitochondrial electron transport chain protein levels, and promotes Drp1 phosphorylation at S616. SBK3 overexpression reversed these anomalies and notably reduced mortality rates in TAC-challenged mice. In this context, our findings support a crucial role for SBK3 in the regulation of mitochondrial homeostasis in cardiomyocytes. Sustaining high expression of SBK3 represents a promising avenue for targeted mitochondrial intervention in hypertrophic heart failure and warrants further investigation.
